# Assessment of cfDNA release dynamics during colorectal cancer surgery

**DOI:** 10.18632/oncotarget.28681

**Published:** 2025-01-21

**Authors:** Mailson Alves Lopes, Maria Elvira Ribeiro Cordeiro, Flávio de Alencar Teles Barreto, Lara de Souza Moreno, André Araújo de Medeiros Silva, Mariana Braccialli de Loyola, Mayra Veloso Ayrimoraes Soares, Joao Batista de Sousa, Fabio Pittella-Silva

**Affiliations:** ^1^Laboratory of Molecular Pathology of Cancer, Faculty of Healthy Sciences, University of Brasília, Federal District, Brasília, Brazil; ^2^Division of Colorectal Surgery, Brasilia University Hospital, Brasília, Brazil; ^*^These authors contributed equally to this work

**Keywords:** colorectal cancer, cfDNA, surgery

## Abstract

Approximately two-thirds of patients with colorectal cancer (CRC) undergo resection with curative intent; however, 30% to 50% of these patients experience recurrence. The concentration of cell-free DNA (cfDNA) before and after surgery may be related to the prognosis of patients with CRC, but there is limited information regarding cfDNA levels at the time of surgery. Here, we analyzed surgical cfDNA release using plasma samples from 30 colorectal cancer patients at three key points during surgery: preoperative (immediately before surgery), intraoperative (during surgery), and postoperative (at the end of surgery). Automated electrophoresis was used to analyze cfDNA concentrations and fragment sizes, which were then correlated with clinical variables. Our findings indicate a significant increase in cfDNA release during and after surgery (2.8- and 2.2-fold higher respectively, *p* < 0.01). Characteristic fragments of cfDNA (<400 bp) predominated at all surgical stages; however, the release of genomic material (>400 bp) was also observed. We found that cfDNA concentration increases during and after surgery in patients over 60 years old (2.9-fold higher intraoperatively than preoperatively and 2.3 folds higher postoperatively than preoperatively, *p* < 0.01); in patients with comorbidities (3.0-fold higher intraoperatively and 2.3-fold higher postoperatively, *p* < 0.01); and in patients with CEA levels >5 ng/mL (3.1-fold higher intraoperatively and 1.3-fold higher postoperatively, *p* < 0.01). Interestingly, cfDNA release during surgery is significantly higher in patients with adverse clinical characteristics. Patients bearing locally advanced tumors or metastasis had a 3.1-fold increase in cfDNA release intraoperatively and 2.4-fold increase postoperatively, *p* < 0.01. cfDNA concentration also increases intraoperatively in patients with a high score of tumor buds (2.6 folds higher, *p* < 0.02), patients with perineural invasion (3.4-fold higher, *p* < 0.02) and in patients with lymphovascular invasion (3.1-fold higher, *p* < 0.05). Furthermore, we observed that cfDNA concentration may rise in correlation with the duration of the surgery, highlighting its potential as a marker of surgical quality. Taken together, our results suggest that in addition to physiological age, comorbidities and unfavorable clinical traits, intense surgical manipulation from the tumor's extent, may result in greater tissue damage and elevated cfDNA release.

## INTRODUCTION

Colorectal cancer (CRC) is a malignant neoplasm originating in the mucosa of the colon or rectum. It is the third most common type of cancer globally, with an estimated approximately 1.9 million new cases and 903,000 deaths projected between 2020 and 2040 [1]. Surgical resection is the best treatment option for patients with colon cancer, but tumor recurrence, both local and distant, is associated with a high risk of cancer-related death. At the time of initial diagnosis, approximately two-thirds of patients with CRC undergo resection with curative intent; however, 30% to 50% of these patients experience recurrence [2, 3].

Anatomopathological staging, the variable most strongly correlated with prognosis, plays a crucial role in guiding treatment decisions. This staging system classifies the disease into four clinical stages, with stage 1 representing the earliest form of the disease and stage 4 indicating advanced, metastatic disease. The TNM system is the most widely used cancer staging system. The latest edition of the Cancer Staging Manual reviewed by the American Joint Committee on Cancer (AJCC), highlights the importance of additional parameters in the TNM system, such as preoperative serum carcinoembryonic antigen (CEA) levels, tumor regression score, lymphovascular and perineural invasion, microsatellite instability, and mutation status of *KRAS*, *NRAS*, and *BRAF* [4–6]. Although these factors are undoubtedly critical, the utility of cell-free DNA (cfDNA) as a prognostic marker in the clinical practice has not yet been widely established.

Recent studies have demonstrated that the concentration of cfDNA before and after surgery may be associated with the prognosis of patients with CRC [7–10]. While these studies suggest the potential use of cfDNA as a surrogate marker for successful tumor removal, there are currently no reports on the dynamics of cfDNA during surgery. It is known that cfDNA is released into the bloodstream after apoptosis and necrosis, containing the molecular signatures of its origin [10]. In the blood of healthy individuals, cfDNA is derived from hematopoietic cells; on the other hand, in individuals with cancer, a fraction of cfDNA is derived from tumor cells [11, 12]. In addition, it has been shown that cfDNA is fragmented non-randomly and that cancer patients have altered fragmentation patterns [12].

Here we aimed to evaluate the dynamics of cfDNA release during surgery in patients with CRC and to analyze the influence of clinical variables on the concentration and fragmentation of cfDNA. Samples were collected at three key points: preoperative (immediately before surgery), intraoperative (during surgery), and postoperative (at the end of surgery).

## RESULTS

### Patients clinical characteristics

To evaluate the correlation of cfDNA dynamics in each time point, we initially performed a descriptive analysis of the main clinical variables of the patients included in the study (Table 1). A total of 9 clinical variables were used in this analysis.

**Table 1:** Descriptive analysis of the categorized clinical variables of patients

Clinical variables		*n*	%
**Age**	<60	12	40.00
	>60	18	60.00
**Sex**	Female	16	53.33
	Male	14	46.66
**Tumor Location**	Rectum	8	26.66
	Colon	22	73.33
**Surgical-Pathological Staging (ypT ypN ypM)**	T3 T4 N- M1	3	10.00
	T3 T4 N- M-	12	40.00
	No T3 T4 N- M-	15	50.00
**Lymphovascular invasion**	Yes	10	33.33
	No	20	66.66
**Perineural invasion**	Yes	9	30.00
	No	21	70.00
**Number of tumor buds**	Not evidenced	1	3.33
	Not informed	5	16.66
	Low score	7	23.33
	Intermediate score	10	33.33
	High score	7	23.33
**Comorbidities**	Yes	17	56.66
	No	13	43.33
**Preoperative carcinoembryonic antigen (CEA)**	<5 ng/ml	13	43.33
	>5 ng/ml	14	46.66
	Not informed	3	10.00

### The release of cfDNA is significantly increased during and after surgery

**Figure 1:**
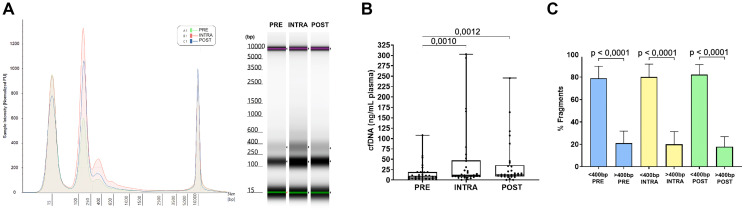
cfDNA release profile, comparison of cfDNA concentrations and comparison of fragment size at each surgical moment. (**A**) Example of the electropherogram and electrophoretic run, with overlays of preoperative, intraoperative, and postoperative moments of the same patient. (**B**) Graphical representation of the mean cfDNA concentrations at preoperative (PRE), intraoperative (INTRA), and postoperative (POST) time points (*n* = 30 patients). The Friedman test was applied to compare PRE, INTRA, and POST (*p* = 0.0055), followed by the Wilcoxon test for PRE vs. INTRA, INTRA vs. POST, and PRE vs. POST. The release of cfDNA during surgery was 2.8-fold higher than before surgery, and the release after surgery was 2.2-fold higher than preoperatively. (**C**) Graphical representation of the mean percentages of fragments <400 bp and >400 bp for PRE, INTRA, and POST. The *T*-test was applied, showing that fragments <400 bp were predominant at all time points.

Initially, cfDNA concentrations extracted from plasma were analyzed, as these values are clinically relevant to the condition of CRC patients [10]. An electropherogram profile and electrophoretic run for preoperative (PRE), intraoperative (INTRA) and postoperative (POST) samples were overlaid for comparison (Figure 1A).

Mean cfDNA concentrations were measured at three surgical moments: preoperative (PRE, before surgery), intraoperative (INTRA, during surgery) and postoperative (POST, after surgery). The release of cfDNA during surgery was 2.8-fold higher than before surgery (*p* < 0.01) (Figure 1B). The release of cfDNA after surgery was 2.2-fold higher than before surgery (*p* < 0.05) (Figure 1B).

### Fragments <400 bp were predominant at all time points

Next, we analyzed the size of the cfDNA fragments at different surgical moments. The size of cfDNA is considered to be less than 400 bp, varying for cfDNA derived from normal and tumor cells, with a predominant peak at 167 bp and 143 bp, respectively [13–15].

Analyzing the fragments classified into <400 bp and >400 bs, we observed that the smaller fragments, attributed to cfDNA, were predominant at all time moments; however, fragmented genomic material was also always recovered (Figure 1C).

### cfDNA concentration correlates with age and comorbidities

**Figure 2:**
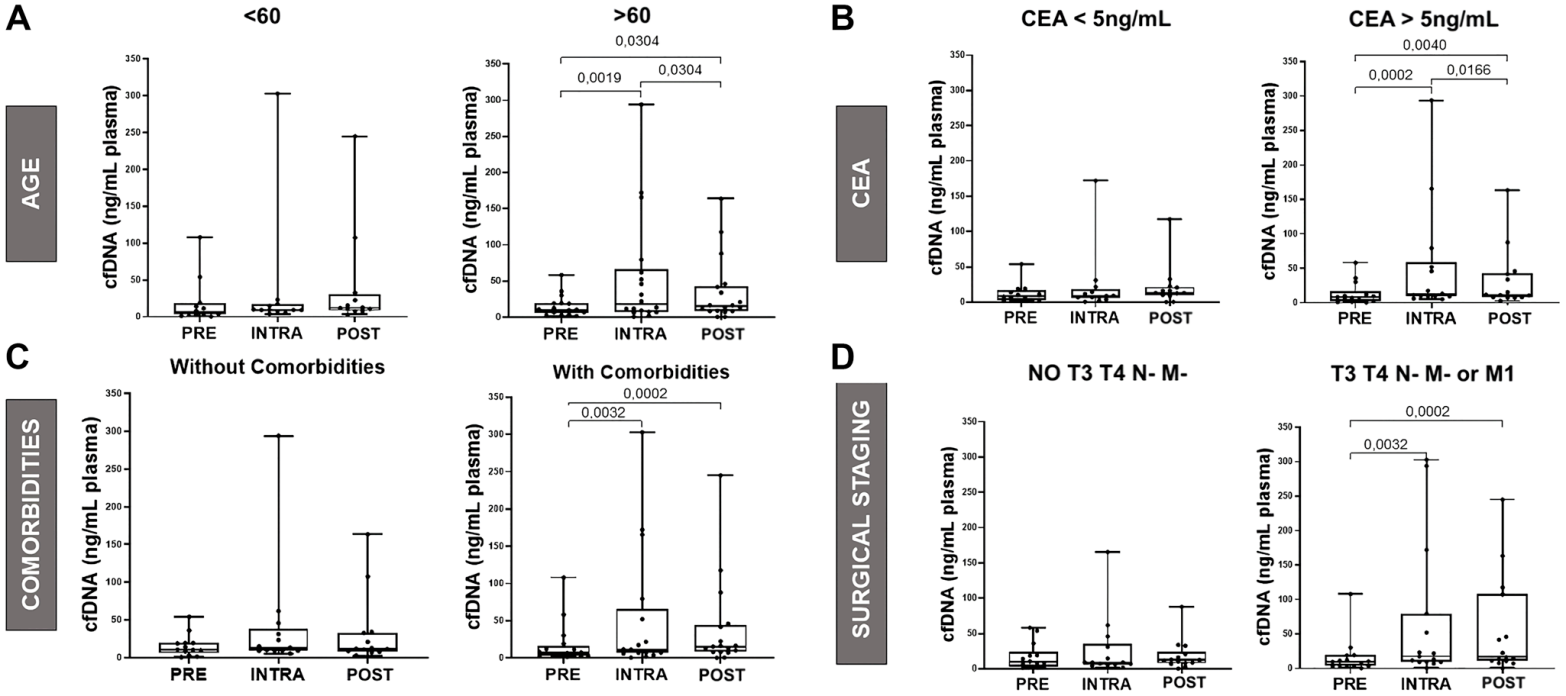
Comparison of cfDNA concentrations with age, CEA (ng/ml), comorbidities and surgical staging. (**A**) Graphical representation of cfDNA (ng/ml plasma) vs. age for PRE, INTRA, and POST time points. The Friedman test was applied (*p* = 0.0111), followed by the Wilcoxon test for PRE vs. INTRA, INTRA vs. POST, and PRE vs. POST. cfDNA release was 2.9-fold higher during surgery compared to preoperative levels, 1.3-fold higher than postoperative levels. (**B**) Graphical representation of cfDNA vs. CEA for PRE, INTRA, and POST. The Friedman test was applied (*p* = 0.0004), followed by the Wilcoxon test for PRE vs. INTRA, INTRA vs. POST, and PRE vs. POST. cfDNA release during surgery was 3.1-fold higher than before surgery and 1.3-fold higher than after surgery. (**C**) Graphical representation of cfDNA vs. comorbidities for PRE, INTRA, and POST. The Friedman test was applied (*p* = 0.0042), followed by the Wilcoxon test for PRE vs. INTRA, INTRA vs. POST, and PRE vs. POST. cfDNA release during surgery was 3.0-fold higher than preoperative levels and 2.3-fold higher than postoperative levels. (**D**) Graphical representation of cfDNA vs. surgical staging for PRE, INTRA, and POST. The Friedman test was applied (*p* = 0.0004), followed by the Wilcoxon test for PRE vs. INTRA, INTRA vs. POST, and PRE vs. POST. cfDNA release during surgery was 3.1-fold higher than both preoperative and postoperative levels, and 2.4-fold higher when comparing postoperative to preoperative levels.

We explored the relation between clinical variables and cfDNA concentrations. We found that in patients over the age of 60 years old, cfDNA release during surgery increased 2.9-fold intraoperatively and remained 1.3-fold higher postoperatively compared to the preoperative (Figure 2A). This difference was not observed in patients with less than 60 years old. A similar trend was observed in patients who had comorbidities. cfDNA release during surgery was 3.0-fold higher than before and 2.3-fold higher in the postoperative compared to preoperative, as shown in Figure 2C (*p* < 0.05).

### cfDNA concentration correlates with Carcinoembryonic Antigen (CEA) and Surgical Staging

Next, we investigated whether CEA levels of patients or the status of their tumor staging correlated with cfDNA release during surgery. We observed that in patients with CEA >5 ng/mL, cfDNA release during surgery was 3.1-fold higher than before surgery and 1.3-fold higher than after surgery (*p* < 0.01), as shown in Figure 2B. In addition, patients with more advanced tumors (classified as T3, T4, N-, M-, or M1) exhibited a 3.1-fold increase in cfDNA release during surgery compared to both before and after surgery, and a 2.4-fold increase in the postoperative period compared to the preoperative period (*p* < 0.001), as shown in Figure 2D.

### cfDNA concentration correlates with the presence of tumor buds and invasion of adjacent tissues

**Figure 3:**
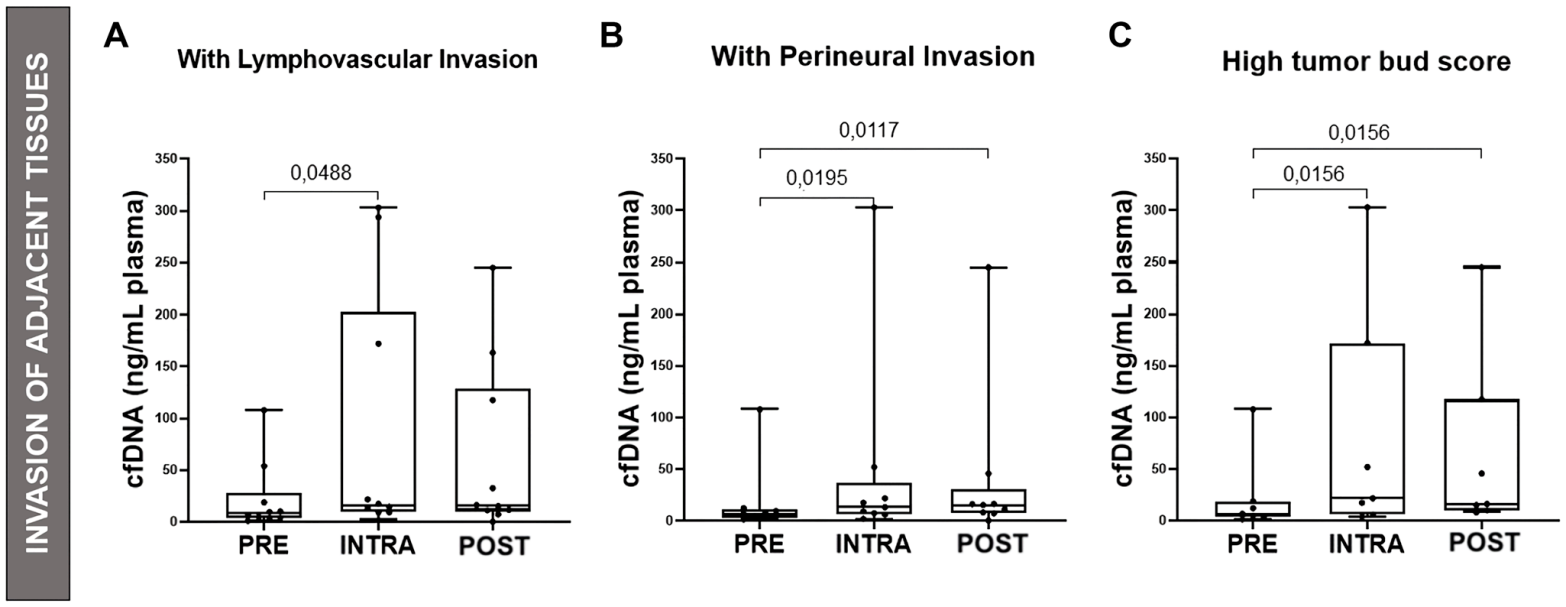
Comparison of cfDNA concentrations with Adjacent Tissue Invasion. Graphical representation of cfDNA (ng/mL plasma) vs. Lymphovascular Invasion (**A**), Perineural Invasion (**B**) and High Tumor Bud Score (**C**). The Friedman test was applied ((A) *p* = 0.0303, (B) *p* = 0.0307, (C) *p* = 0.0012), followed by the Wilcoxon test for PRE vs. INTRA, INTRA vs. POST and PRE vs. POST. In patients with lymphovascular invasion, cfDNA release was 3.1-fold higher during surgery than before surgery. In patients with perineural invasion, cfDNA release was 3.4-fold higher during surgery and 2.6-fold higher in the postoperative period compared to the preoperative period. And in patients with a high score of tumor buds, the release of cfDNA was 3.5-fold higher during surgery and 2.6-fold higher in the postoperative period compared to the preoperative period.

We subsequently analyzed the correlation between cfDNA levels and characteristics related to adjacent tissue invasion. In patients with a high tumor bud score, cfDNA release was 3.5-fold higher during surgery compared to pre-surgery levels, and 2.6-fold higher in the postoperative period compared to the preoperative period (*p* < 0.02). Similarly, in patients with perineural invasion, cfDNA concentration was 3.4-fold higher during surgery and 2.6-fold higher in the postoperative period compared to pre-surgery (*p* < 0.02). Furthermore, in patients with lymphovascular invasion, cfDNA release during surgery was 3.1-fold higher compared to preoperative levels (*p* < 0.05), as shown in Figure 3.

### cfDNA concentration varies according to the time of surgery and disease severity parameters

**Figure 4:**
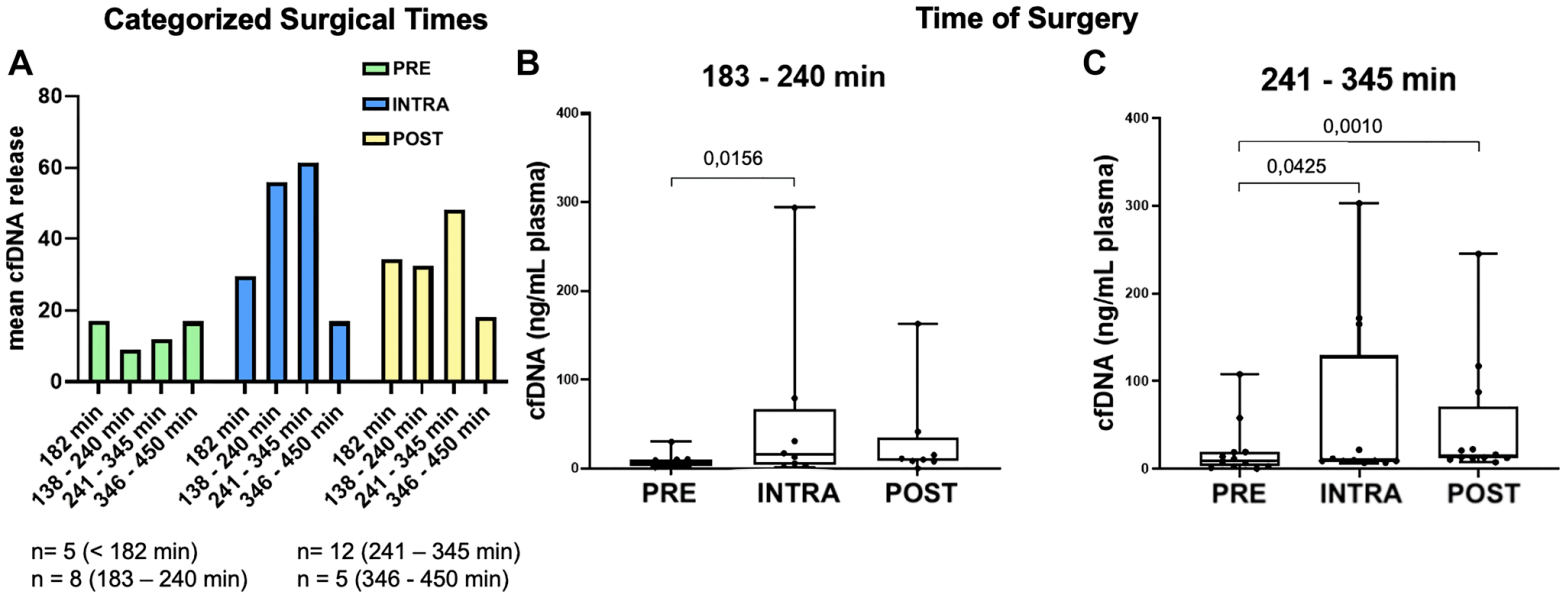
Comparison of cfDNA concentrations with duration of surgery. (**A**) Graphical representation of overall cfDNA release (ng/mL plasma). (**B**) Graphical representation of cfDNA release in surgeries lasting 183–240 minutes. (**C**) Graphical representation of cfDNA release in surgeries lasting 241–345 minutes. The Friedman test was applied ((B): *p* = 0.0469, (C): *p* = 0.0131), followed by the Wilcoxon test for comparisons between PRE vs. INTRA, INTRA vs. POST, and PRE vs. POST. In surgeries lasting 183–240 minutes, cfDNA release during surgery was 6.1-fold higher compared to preoperative levels, while in surgeries lasting 241–345 minutes, it was 5.1-fold higher. It was observed a significant increase in cfDNA release during surgeries lasting 183–240 minutes and 241–345 minutes, reaching 6.1-fold higher and 5.1-fold higher, respectively.

Finally, we analyzed the influence of surgical time on cfDNA release. We stratified patients in four groups according to the time of surgery and compared the cfDNA release in each group, before, during and after surgery (Figure 4A). We observed a significant increase in cfDNA release during surgeries lasting 183–240 minutes and 241–345 minutes, reaching 6.1-fold and 5.1-fold higher levels, respectively (Figure 4B, 4C).

**Figure 5:**
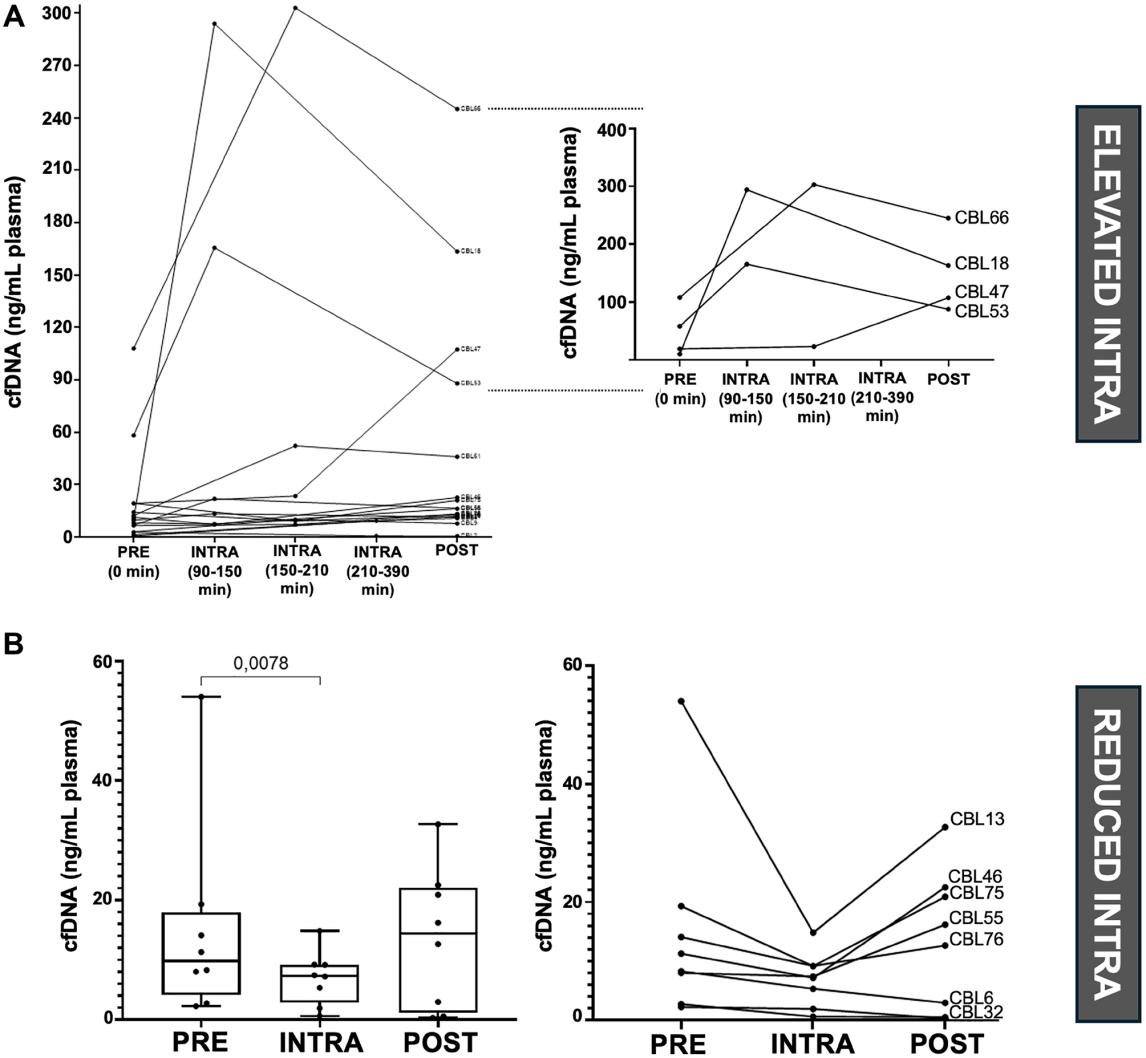
Comparison of cfDNA concentrations with blood sampling time (*n* = 16), analysis of elevated (*n* = 4) and reduced (*n* = 8) cfDNA concentrations during surgery. (**A**) Graphical representation of the overall release of cfDNA at each blood sampling time during surgery for each patient on the left. On the right, an expanded view showing the elevated release of cfDNA for each patient. (**B**) Graphical representation of cfDNA release in patients in whom a reduction in cfDNA was observed during surgery. On the left, general graphic. Friedman’s test was applied (*p* = 0.0469), followed by the Wilcoxon test for comparisons between PRE vs. INTRA, INTRA vs. POST and PRE vs. POST. On the right, graphical representation of cfDNA release per patient.

We also compared cfDNA concentrations in blood samples from 16 patients collected at different time points during surgery. Notably, the three patients with locally advanced tumors (highlighted in Figure 5A: CBL66, CBL18, and CBL47) exhibited the highest levels of cfDNA release intraoperatively. In contrast, a group of patients with reduced cfDNA concentrations during surgery predominantly consisted of those with less severe cases, characterized by early-stage tumors, CEA levels <5 ng/ml, and no evidence of tissue invasion (Figure 5B).

## DISCUSSION

cfDNA and ctDNA, the fraction of cfDNA derived from cancer cells, can be analyzed through liquid biopsy, a promising technique for various types of cancer [16, 17]. Its detection has become a key factor in cancer diagnosis, personalized therapeutic interventions, treatment monitoring, and prognosis, as cfDNA concentrations are often elevated in cancer patients [12, 18]. Interestingly, high cfDNA levels in cancer patients do not exclusively originate from cancer cells, but also from immune system cells, particularly neutrophils, suggesting that cfDNA release may reflect a broader systemic effect [12]. Furthermore, environmental and physiological factors have been shown to influence both the amount and fragmentation profile of cfDNA [19].

Given the complexity of cfDNA release dynamics and its expanding clinical relevance, it is essential to investigate these influencing factors during surgery and account for patient-specific variables that may impact cfDNA release patterns.

During surgery, we observed a significant increase in cfDNA release between the preoperative, intraoperative, and postoperative periods, likely due to tissue manipulation, especially during surgery when manipulation is most intense.

Analysis of DNA fragments revealed a statistical difference in fragment sizes at each surgical stage. While fragments characteristic of cfDNA (<400 bp) were predominant, larger fragments (>400 bp), potentially representing genomic DNA, were present at all stages.

Several studies have suggested that cfDNA integrity and fragmentation are highly variable characteristics and may reflect the altered genomic structure in cancer patients [17, 20]. Smaller fragments of cfDNA are often associated with apoptosis and necrosis events, while larger fragments can originate from various physiological mechanisms, including hematological and biochemical processes [13, 21]. These findings highlight the possibility of high levels of non-cancerous cfDNA. This hypothesis is consistent with our study suggesting a correlation between fragments >400 bp and surgical manipulation.

In addition, it has been observed that samples with undetectable ctDNA can have highly fragmented cfDNA profiles. This phenomenon was particularly observed in samples from patients who had recently undergone surgery. These observations suggest that cfDNA size distributions can be misleading in certain contexts and highlight the importance of examining cfDNA characteristics at different time points since diagnosis [22].

We observed that cfDNA release during surgery varies across specific patient groups, including those over 60, those with comorbidities, CEA >5 ng/mL, and patients with advanced tumors (T3, T4, N-, M-, or M1). cfDNA levels have been linked to various health conditions, including comorbidities and age-related changes [23]. It has been reported that cfDNA concentrations increase in the elderly and may serve as a biomarker of aging [24]. In our study, intraoperative cfDNA release was higher in patients over 60 and in those with comorbidities, with the majority of elderly patients also having comorbidities based on medical records.

CEA is a recommended tumor marker for colorectal cancer. It has been reported that CEA levels typically normalize after surgery, but persistently high postoperative levels (>5 ng/mL) are associated with a poor prognosis, including metastasis and recurrence [25]. In this study, patients with preoperative CEA >5 ng/mL exhibited greater cfDNA release during surgery compared to those with CEA <5 ng/mL. Additionally, patients with locally advanced tumors or distant metastases had higher intraoperative cfDNA release compared to those with early-stage disease (N0, T3, T4, N-, M-). This increased cfDNA release may result from more extensive surgical manipulation due to tumor size, leading to greater tissue damage.

We also found that cfDNA release may be linked to adjacent tissue invasion. Patients with high tumor bud scores, perineural invasion, or lymphovascular invasion showed significantly elevated cfDNA levels during and after surgery. Given that tumor budding is strongly associated with regional or distant metastasis, and both perineural and lymphovascular invasion indicate a poor prognosis [26–29], our findings suggest that increased cfDNA release during surgery, along with factors such as age, comorbidities, staging, and CEA levels, may serve as an indicator of poor prognosis. This hypothesis is supported by the observation that early-stage patients with a good prognosis exhibited a decrease, rather than an increase, in cfDNA release during surgery.

In addition, we observed that cfDNA levels varied with the duration of surgery, showing a marked increase during operations lasting 3 to 6 hours, particularly in patients with locally advanced tumors. These results suggest that cfDNA may serve as a marker of surgical quality, though further investigation is needed.

In summary, this is the first study to report on the dynamics of cfDNA release during surgery in CRC patients. Our results demonstrate a significant increase in cfDNA release during and after surgery, with characteristic cfDNA fragments (<400 bp) being predominant at all surgical stages, alongside genomic fragments (> 400 bp). Intraoperative cfDNA release was especially notable in patients over 60, those with comorbidities, CEA levels >5 ng/mL, locally advanced tumors, metastases (T3, T4, N-, M-, or M1), or features indicating tissue invasion. Conversely, patients with early-stage disease and a favorable prognosis often showed reduced cfDNA release during surgery, while those with advanced disease had higher cfDNA levels and poorer outcomes.

Although this study has provided significant information on the dynamics of cfDNA release during surgical procedures, there are some limitations to consider. These include the relatively small sample size, potential biases in sample recovery and fragment size considerations.

## MATERIALS AND METHODS

### Patient cohorts and sample processing

Blood samples were obtained from patients who provided informed consent under the study approved by the Institutional Ethics Committee (CAAE number 65822222.4.0000.0030). The samples and data were collected from 30 patients diagnosed with CRC who underwent surgery at the Coloproctology Division of the University Hospital of Brasília (HUB). Inclusion criteria were: (i) a biopsy-confirmed malignant tumor; (ii) an inclusion period from February 2023 to February 2024; (iii) collection of the three samples during surgery (preoperative, intraoperative, and postoperative); (iv) a minimum of 4 ml of plasma after blood sample preparation.

### Sample collection and preparation

We collected blood samples in three different points: (i) preoperative, with the patient already in the operating room before induction of anesthesia; (ii) intraoperative, after removal of the surgical specimen; and (iii) postoperative, at the end of the surgery, with the patient still in the operating room. Plasma was separated from blood collected in EDTA-Na_2_ tubes within 2 hours after blood was drawn. Tubes were centrifuged at 2000 × g for 10 minutes at 4°C and the supernatant (plasma) was recovered and stored at –80°C until cfDNA extraction. Before extraction, the plasma sample was centrifuged at 16000 g for 10 min at 4°C to remove cell debris [13].

### Extraction of cfDNA

The cfDNA was extracted using the MagMAX Cell Free Total Nucleic Acid Isolation Kit (ThermoFisher), according to the manufacturer’s manual. The amount, quality and fragmentation of the cfDNA obtained was measured in an automated electrophoresis system using TapeStation equipment (Agilent).

### Analysis of clinical data and molecular data

We analyzed the clinical data from the patients’ medical records, along with data on cfDNA concentration and fragment size (with fragments <400 bp being attributed to cfDNA and longer fragments >400 bp being attributed to genomic material) using GraphPad Prism Software (GraphPad Software Inc.). For statistical analysis of cfDNA concentration and clinical variables, considering the distribution profile of the data, as well as the requirement for a paired model, we used the Friedman test to evaluate all the time points and the Wilcoxon test to compare each time point analyzed. For the analysis of fragments, we used the Student’s *t*-test, considering the distribution of fragmentation data higher or lower than 400 bp. The specific tests applied are detailed in the figure legends for each result. Differences were considered statistically significant if *P*-values were less than 5% (*p* < 0.05).
